# Trend analysis of hepatitis B and C among patients visiting health facility of Tigrai, Ethiopia, 2014–2019

**DOI:** 10.1186/s12876-023-02807-2

**Published:** 2023-05-19

**Authors:** Haftom Legese, Brhane Berhe, Gebre Adhanom, Tsega Kahsay, Aderajew Gebrewahd, Guesh Gebremariam, Fitsum Mardu, Kebede Tesfay, Haftay Gebremedhin, Hadush Negash

**Affiliations:** 1 Department of Medical Laboratory, College of Health Sciences, Adigrat University, Adigrat City, Tigrai Ethiopia; 2 Department of Public Health, College of Health Sciences, Adigrat University, Adigrat City, Tigrai Ethiopia

**Keywords:** Ethiopia, Hepatitis B, Hepatitis C virus, Tigrai, Trend

## Abstract

**Background:**

Hepatitis B and C viruses are the major public health concerns of the globe. The two hepatotropic viruses share common modes of transmission and their co-infection is common. Despite the provision of an effective prevention mechanism, the infections caused by these viruses remain a significant problem worldwide, particularly among developing countries like Ethiopia.

**Methods:**

This institutional-based retrospective study was conducted between January 2014 December and December 2019 from documented laboratory logbooks of Adigrat general hospital serology laboratory, Tigrai, Ethiopia. data were collected and checked for completeness on a daily based, coded, entered, and cleaned using Epinfo version 7.1, exported and analyzed using SPSS version 23. Binary logistic regression analysis and Chi-square test (X^2^) assessed the association between dependent and independent variables. The corresponding variables with a P-value (P < 0.05) and 95% confidence interval were considered statistically significant.

**Results:**

Out of 20,935 clinically suspected individuals, 20,622 were given specimens and tested for hepatitis B and C viruses with total completeness of 98.5%. The overall prevalence of hepatitis B and hepatitis C virus was found to be 3.57% (689/19,273) and 2.13% (30/1,405), respectively. The positivity rate of the hepatitis B virus was 8.0% (106/1317) and 3.24% (583/17,956) among males and females, respectively. Additionally, 2.49%( 12/481) of males and 1.94% (18/924) of females were positive for hepatitis C virus infection. The overall prevalence of co-infection for both hepatitis B and hepatitis C virus was 7.4% (4/54). Sex and age were significantly associated with hepatitis B and C virus infection.

**Conclusions:**

The overall prevalence of hepatitis B and C is low intermediate according to the WHO criteria. Although there was a fluctuating trend of hepatitis B and C through the years 2014–2019, the result shows moreover declining trend. Both hepatitis B and C share similar routes of transmission and affect all age categories but males were more highly affected than females. Therefore, awareness creation of the community about the methods of transmission, education about prevention, and control of hepatitis B and C virus infection, and improving coverage of youth-friendly services in health facilities should be strengthened.

## Background

Viral hepatitis (hepatitis A, B, C, D, and E) becomes a major public health concern, infecting millions of people globally [1, 2]. Approximately 1.4 million deaths are caused by viral hepatitis each year [3, 4], HBV and HCV are responsible for about 96% of these fatalities [5]. Hepatitis B virus (HBV) and hepatitis C virus (HCV) infections are causing serious health problems in the world. Globally, 257 and 10 million people were carrying HBV and HCV infections, respectively. Of these, 80 million have active viraemic infections [6, 7]. Without treatment, up to 30% of chronic HBV and 20% of chronic HCV cases develop liver cirrhosis and hepatocellular carcinoma, which are the sixth most common cancer and the third cause of cancer-associated deaths worldwide [8–11]. The prevalence of HBV infection varied from high (≥ 8%) to intermediate (2–7%) and low (*<* 2%). Similarly, HCV infection is high (*>* 3.5%), moderate (1.5–3.5%), and low (*<* 1.5%) prevalence[12].

Even thoughthe burden of HBV and HCV infection has a worldwide distribution, the vast majority of infected persons reside in low and middle-income countries [13]. Africa has one of the highest burdens of disease estimated 60 million people are infected. The prevalence of HBsAg is 6·1%, withabout 87,890 deaths annually in Africa. However, the Sero-prevalence differs depending on sex, ethnicity, and rural residence [14–16].

The mode of transmission for HBV and HCV is through horizontal transmission, carrying out healthcare procedures, using contaminated instruments, using unsafe practices (unsafe sexual practices, reuse of needles and syringes), and mother-to-child transmission [17]. Co-infection with the two viruses is common [18].

The true public health magnitude and impact of hepatitis epidemics are poorly understood in African countries like Ethiopia. Because national and sub-national data are insufficient, and HBV and HCV surveillance programs are weak making it is difficult to plan for focused action and prioritize the allocation of resources in Ethiopia [19].

Even though patients who are clinically suspected of HBV and HCV have been shown to have a high prevalence of hepatitis infection, awareness about the magnitude of this condition is limited among health care workers and caretakers [19]. Hence, thus this study was conducted to determine the extent of hepatitis infection among patients attending Adigrat General Hospital.

## Material and Methods

### Study design, area, and period

An institutional-based retrospective study was conducted at Adigrat General Hospital from January 2014 to December 2019. The Hospital is found in Adigrat city eastern zone of Tigrai regional state, located 891 km from Addis Ababa (the capital city of Ethiopia) and 104 km from Mekelle city (capital of Tigrai regional state). The city is a zonal administrative of Eastern Tigrai, Ethiopia with an estimated total population of 76,400 [20]. Adigrat General Hospital is one of the governmental Hospitalsof Tigrai with catchments of 6 woredas. The Hospital is having about 120 beds with a total of 209 healthcare professionals and 132 administrative staff. Currently, it served as a teaching and referral hospital, serving more than 1,000,000 populations with an average annual client flow of 131,125 people.

### Study population

All clients who presented to the outpatient departments and those that were admitted irrespective of their HIV status and had a request for HBV and HCV screening gave blood and recorded in the laboratory logbook at the Adigrat General Hospital from January 2014 to December 2019.

### Sample size

All of the 20,622 HBV and HCV-suspected individuals who were tested in the Adigrat General Hospital laboratory from January 2014 to December 2019 that fit the inclusion criteria were the total sample size for this study.

### Inclusion criteria

Data showed a HBV and HCV statuses, years, ages and sex were included in this study.

### Exclusion criteria

Missed data in the inclusion criteria were excluded.

### Definition of terms

Hepatitis B is an infection caused by the hepatitis B virus (HBV). For most people, hepatitis B is short term, also called acute, and lasts less than six months. But for others, the infection becomes chronic, meaning it lasts more than six months. Hepatitis C is an inflammation of the liver caused by the hepatitis C virus. The virus can cause both acute and chronic hepatitis, ranging in severity from a mild illness to a serious, lifelong illness including liver cirrhosis and cancer [21].

### Data collection tool

Data were collected from HBV and HCV-suspected clients attending at Adigrat General Hospital for the period of spanning January 2014 through December 2019 from the logbooks by trained six medical laboratory technologists. Relevant parameters (age, sex, year of screening and status HBV and HCV) were collected. Further data on different factors associated with.

HBV and HCV infection were problematic because the logbook contained small variables.

### Handling and tracking of missing data

In case of missing data from the laboratory logbooks were inspected and tracked to be included in the study. However, incomplete laboratory logbooks were excluded and consulted to the head department of the laboratory of Adigrat hospitals.

### Laboratory methods

The HBsAg and anti-HCV antibody was requested for suspected clients attending at Adigrat General Hospital. The presence of HBsAg and anti-HCV antibodies in serum/plasma was detected using a One Step Cassette Style HBsAg test kit (ACON Laboratories, Incorporated San Diego, California, USA). The HBsAg test strip has a relative sensitivity 100% (98.02-100%) and Relative Specificity: 100% (98.81-100%, and Wondfo one step hepatitis C virus test, (Wondfo Biotech Co., Ltd., Guangzhou, PRC). The anti-HCV antibody test strip has with sensitivity 99% and a specificity of 99.8% by following the Manufacturer’s instructions.

#### Strength

The strength of ACON HBsAg and Wondfo anti-HCV antibody one step Rapid Test Cassette (Serum/Plasma) is it has high sensitivity and specificity High reproducibility, Consistent and reliable performance, Easy to use, and all necessary reagents provided.

#### Limitation

ACON HBsAg Rapid Test Cassette (Serum/Plasma) is for in vitro diagnostic use only. This test should be used for the detection of HBsAg in serum or plasma specimen. Only indicate the presence of HBsAg in the specimen and should not be used as the sole criteria for the diagnosis of Hepatitis B viral infection. As with all diagnostic tests, all results must be considered with other clinical information available to the physician. Rapid Test cannot detect less than 0.79 ng/mL of HBsAg in specimens [22]. Wondfo anti-HCV antibody one step Rapid Test has been developed for testing whole blood /serum/plasma samples only. The performance of the test using other specimens has not been validated. This test is a qualitative screening assay; it is not determining the quantitative concentration of HCV antibody [23].

#### Quality control

To assure the quality of data, we first checked the completeness of the HBV and HCV serology laboratory logbooks. A properly designed data collection format sheet was prepared and used for HBV and HCV data recording. Then data were collected from laboratory logbooks and client charts and records which were stored from 2014 to 2019 in the laboratory of the Hospital. Data were extracted by trained data collectors and were adequately informed about the data collection process. Each day the collected data were reviewed for completeness and consistency of the information. Furthermore, the laboratory technologists of the hospital were well-trained and had more than 6-year work experience. The hospital is strict when it comes to the use of standard operating producers and the professionals run positive and negative controls and checked for expired date before they process patients test.

#### Ethical clearance

All procedures performed in this study were performed in accordance with the guideline and regulations approved by the Research Ethics Review Committee (RERC) of the Tigrai regional research institution and ethical clearance (Consent Ref Number THRI/4031/0390/19 approval dated 25/11/2019). The official letter was obtained from Tigrai Regional Health Bureau (Consent Ref Number TRHB/RCSH 121/1418/2019 approval dated 30/11/2019 to Adigrat Hospital administration.

#### Data processing and analysis

Data were cleaned, entered, and analyzed using Statistical Package for Social Sciences (SPSS) version 23 database. Descriptive statistics like frequency, percentage, measures of central tendency, and measures of dispersion were carried out. Missing values were analyzed by using multiple imputation techniques. Then the data were presented using frequencies tables and figures. Binary logistic regression analysis and Chi-square test (X^2^) were used to calculate the odds ratios (OR); Crude Odds Ratio (COR) and Adjusted Odds Ratio (AOR) to ascertain the degree of association between the dependent and independent variables. The multi-co-linearity test was carried out to see the correlation between independent variables. The corresponding variables with a P-value < 0.05 with 95% confidence interval were considered statistically significant.

## Result

### Socio-demographic characteristics of study subjects

Of the total 20,935 requested samples, 20,622 (98.5%) patients provided complete data and included in the study. Most of them, 18,862 (91.5%) were females. A higher frequency of study participants 8,954 (43.4%) were aged from 25 to 34 years. mean and standard deviation was 28 (± 9.54SD) [Table 1].

**Table 1:** Socio-demographic characteristics of study participants at Adigrat General Hospital from January 2014 to December 2019

Variables	Frequency	Percentage (%)
**Sex**		
Male	1760	8.5
Female	18,862	91.5
**Age**		
0–14	419	2.0
15–24	7468	36.2
25–34	8954	43.4
35–44	2705	13.1
45–54	421	2.0
55–64	234	1.1
≥ 65	421	2.0

### Prevalence of HBV, HCV and HBV-HCV co-infection

The overall prevalence of HBsAg and anti-HCV were 3.57% (689/19,273) and 2.13% (30/1,405), ,respectively. Among the study participants, the positivity rate of HBsAg was 106/1317 (8.0%) among males and 583/17,956 (3.24%) females. Additionally, 12 (2.49%) of males and 18 (1.94%) females were positive for hepatitis C, respectively. Table 2 showed the prevalence of co-infection for HBV and HCV was 4/54 (7.4%). Hepatitis B virus was predominantly higher among individuals aged ≥ 65 years old (6.27%) followed by 45–54 years old (4.79%). On the other hand, the prevalence of hepatitis C virus was high frequency among the age group of 45–54 years old (7.52%) followed by 55–64 years old(3.84%). Moreover, a high percentage of HBV and HCV were seen in the year of 2014 [Table 2].

**Table 2:** Detection rates of HBV, HCV, and HBV-HCV co-infection among study participants at Adigrat General Hospital from January 2014 to December 2019

Year	HBV suspected N (%)	HBV Positive N (%)	HCV Suspected N (%)	HCV Positive N (%)	Both HBV and HCV suspected N (%)	Both HBV and HCV Positive N (%)
**Sex**						
Male	1317	106(8.0)	481	12(2.49)	36	1(2.63)
Female	17,956	583(3.24)	924	18(1.94)	18	3(16.67)
**Age**						
0–14	364	8(2.19)	56	1(1.78)	1	1(100.0)
15–24	7085	220(3.1)	404	4(0.99)	21	1(4.76)
25–34	8526	317(3.71)	447	8(1.78)	19	1(5.26)
35–44	2474	103(4.16)	235	6(2.55)	4	0(0.0)
45–54	334	16(4.79)	93	7(7.52)	5	1(16.67)
55–64	187	6(3.2)	52	2(3.84)	4	0(0.0)
≥ 65	303	19(6.27)	118	2(1.69)	0	0(0.0)
**Year**						
2014	989	91(9.2)	125	7(5.6)	41	0(0.0)
2015	1845	66(3.57)	79	10(12.65)	10	3(30.0)
2016	936	27(2.88)	165	0(0.0)	2	0(0.0)
2017	3900	71(1.82)	284	7(2.46)	1	1(33.34)
2018	4351	148(3.4)	136	1(0.73)	0	0(0.0)
2019	7252	286(3.94)	616	5(0.81)	0	0(0.0)

### Factors associated with hepatitis B virus infection

Bivariate and multivariate logistic regression analyses were performed to assess the association between dependent and independent study variables. According to the bivariate analysis, being female and age showed an association with hepatitis B virus infection and transported to multivariate analysis. Accordingly, in multivariate analysis, being female was negatively associated with hepatitis B virus infection (AOR = 0.439, 95% CI: 0.348,0.553, p = 0.001) and age between 15 and 24 (AOR = 2.320, 95%, CI: 1.125, 4.788,p = 0.023), 25–34(AOR = 2.877, 95% CI: 1.397, 5.924, p = 0.004), 35–44 (AOR = 2.927, 95% CI: 1.401, 6.115, p = 0.004) and 45–54 (AOR = 2.493, 95% CI: 1.051, 5.912, p = 0.038) were significant associated with hepatitis B virus infection [Table 3].

**Table 3:** Factors associated with hepatitis B among study participants at Adigrat General Hospital from January 2014 to December 2019

Variables	Positive for HBV		COR^a^ (95% CI)	p-value	AOR^b^ (95% CI)	p-value
	Yes	No				
**Sex**						
Male	106	1211	1		1	
Female	583	17,373	0.498(0.402,0.616)	0.001	0.439 (0.348,0.553)	0.001*
**Age**						
0–14	8	356	1		1	
15–24	220	6865	1.559(0.765, 3.179)	0.222	2.320(1.125, 4.788)	0.023*
25–34	317	8209	1.886(0.928, 3.830)	0.079	2.877(1.397, 5.924)	0.004*
35–44	103	2371	2.034(0.983, 4.207)	0.056	2.927(1.401, 6.115)	0.004*
45–54	16	318	2.030(0.859, 4.795)	0.107	2.493(1.051, 5.912)	0.038*
55–64	6	181	1.352(0.463, 3.945)	0.581	1.435(0.491, 4.196)	0.509
≥ 65	19	284	2.428(1.051, 5.610)	0.038	2.237(0.966, 5.177)	0.060

Key: ^a^(COR = Crude odds ratio); ^b^(CI = Confidence interval); ^c^(AOR = Adjusted odds ratio); 1(referent).

### Factors associated with hepatitis C virus infection

Chi-square test (X^2^) showed that gender (p < 0.001), and age (p < 0.001), were statistically significant with hepatitis C virus infection [Table 4].

**Table 4:** Factors associated with hepatitis C among study participants at Adigrat General Hospital from January 2014 to December 2019

Variables	Positive for HCV		Chi-square test (X^2^)	p-value
	Yes	No		
**Sex**				
Male	12	469	38.105	0.001*
Female	18	906		
**Age**				
0–14	1	55	85.587	0.001*
15–24	4	400		
25–34	8	439		
35–44	6	229		
45–54	7	86		
55–64	2	50		
≥ 65	2	116		

### Trend prevalence of HBV and HCV infections

**Fig. 1:**
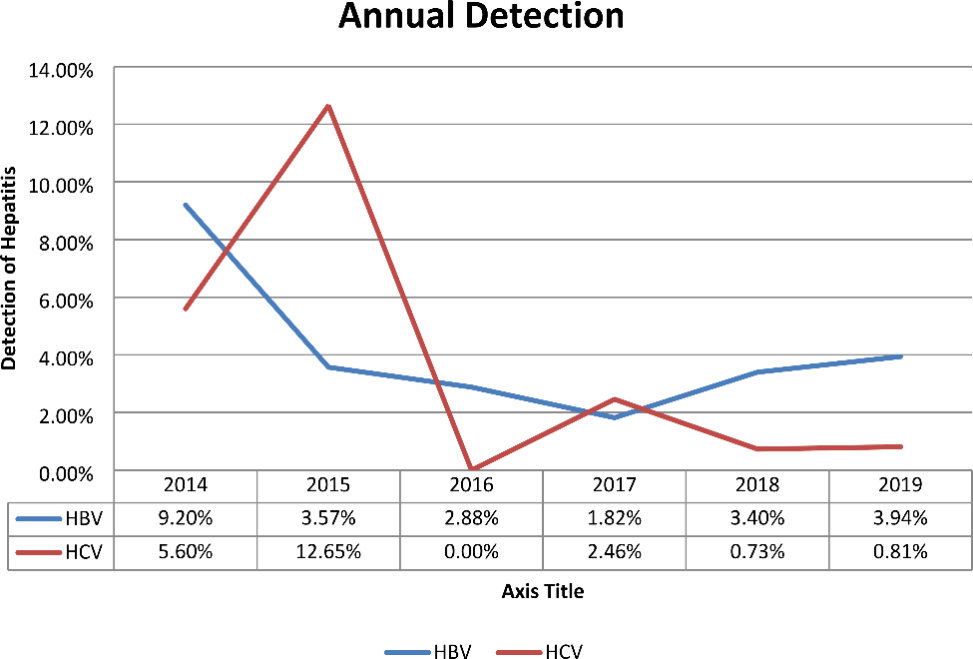
Trend prevalence of HBV, HCV among study participants at Adigrat General Hospital from January 2014 to December 2019

The prevalence of HBV and HCV was relatively fluctuating from year to year. The trend line analysis showed that high prevalence of hepatitis B virus was observed in 2014, 9.2% but it decreased from 2014 to 2017 from 9.2 to 1.82%. On the other hand, HBV increased from the year 2018 (3.40%) to the year 2019 (3.94%). Furthermore, the trend prevalence of hepatitis C virus observed a considerable fluctuation from year to year. Prevalence was 5.60% in 2014, 12.65% in 2015, 0.0% in 2016, 2.46% in 2017, 0.73% in 2018, and 0.81% in 2019 (Fig. 1).

## Discussion

According to the World Health Organization, viral hepatitis plays a significant role in the burden of chronic diseases. Infections with HBV and HCV cause liver cirrhosis and primary liver cancer worldwide [24].

In this study, the overall prevalence of HBV and HCV infections among suspected clients in Adigrat Hospital was 3.57% and 2.13%, respectively. This study was lower compared with similar studies conducted in Ethiopia, Gondar 14.6% HBV and 12.4% HCV [19], Hawassa 9% HBV and 5.5% HCV [25], Ghana 6.94% HBV [26]and, Nigeria 13.60% and 16.6% for HBV and HCV, respectively [27]. The possible explanation for this difference could be due to the hyperendemic in Ghana and Nigeria, variation in methodology, the difference in the population studied the level of awareness the method of transmission. Additionally, a study conducted in Egypt on intravenous drug addicts showed 24% of HBsAg positive and 33% were positive for anti-HCV. This study was higher compared with the present findings. This might be due to differences in study subjects who were highly vulnerable to hepatitis infection due to shared use of needles and more parenteral exposure [28]. However, compared to our study low prevalence was reported in Iran 0.13% for HBV and 0.06% for HCV [29]. This might be attributed to the study participants in Iran were blood donors they are a self-selected group at a lower risk of infectious diseases and the exclusion of those individuals having signs and symptoms of the disease [29].

A co-infection rate of 7.4% (4 /54) was reported in this study. This finding is higher than with the study reported in Ethiopia, Gondar 2% [19], this is due to the difference in sample size. However, it was lower compared with the study done in Nigeria 8.3% [30]. This might be due to differences in the study period, increasing awareness of the population about the importance of screening, and the sample size of the study participant [30].

Among the male participants, 106 (8.0%) were seropositive for HBsAg and 12 (2.49%) were positive for HCV antibody compared to the 583 (3.24%) and 18 (1.94%) positive HBsAg and HCV antibodies respectively among the female study participants. This is consistent with the study conducted in Nigeria [27]. The higher prevalence in men might be due to the sharing of sharps materials such as nail cutters and barbing clippers and injectable drug usage is also more prevalent in males than in females [28].

In this study, sex was significantly associated with HBV infection. Female study participants were fourfold less likely to be infected by the hepatitis B virus infection compared to male study participants. This finding is in line with previous studies conducted in Gondar [19], Brazil [31], Pakistan [32] [33], and, Ghana [34]. This may be due to drug use and occupational exposure compared to women. In contrast, a higher prevalence of hepatitis B virus (HBV) was observed among females compared to their male counterparts [26].

The present study showed that age was significantly associated with HBV. The study participant’s age groups from 15 to 24, 25–34, and 35–44 were three times more likely to have HBV infection. This finding is consistent with the study reported from, Gondar, Ethiopia [19], and Nigeria [27]. This might be due to tattooing, drug use, those age groups that are sexually active in nature, alcoholism, and occupational exposure. Hence, this may be an implication to take action such as increased resource mobilization for prevention and control of this infection, improve awareness of this group about the availability of vaccination, increase coverage of youth-friendly service in the health facilities, actions to prevent transmission of the virus, guidelines for screening, diagnosis. Moreover, age is thought to be strongly associated with hepatitis B infection those individuals aged 45–54 were also 2.4 more likely to acquire HBV infection. This finding was also supported by studies conducted in Brazil [31] and [27]. This might be due to the increased practice of dental extraction out of health institutions with unhygienic conditions and without proper sterilization[25].

In the current study, age and sex were statistically significant with hepatitis C infection. This is agreed with finding reported from Gondar, Ethiopia [19], and Nigeria [27].

In our study, the prevalence of hepatitis B virus infection showed a significant upward in 2014. Even though a gradual decline was shown from 2015 to 2017, small peaks were observed from 2018 to 2019. The decrease in the prevalence of the hepatitis B virus may be attributed to the increasing awareness of the population about the method of transmission and prevention, and the control mechanism of the infection. Whereas, small peaks might be due to increasing awareness towards screening from 3897 to 2017, 4498 in 2018, and 7538 in 2019 and increased awareness of the hepatitis B virus which already exists in the community.

Additionally, the prevalence of hepatitis C virus infection was varied noticeably in different years. Trend of HCV prevalence was observed among clinically suspected individuals in2014, 5.6% then it was increased to 12.65% in 2015 and lowered to 0.0% 2016. On the other, a small fluctuation was shown for HCV from 2017, 2.46%, 2018, 0.73%, and 2019, 0.81%. During the subsequent period studied, it fluctuated, peaking attributed to increasing in HCV screening for the clinically suspected patients.

## Limitations

Since the study is secondary data from the logbook data incompleteness, lack of the associated risk factor, and poor document retention system were a limitation of this study.

Furthermore, for future researcher detection and introduction of PCR-based screenings are important.

## Conclusions

The overall trend prevalence of hepatitis B and C viruses among patients suspected and referred to Adigrat Hospital serology laboratory were 3.57% and 2.13%, respectively. Despite the fluctuation in the trend analysis of hepatitis B and C we observed a bit of elevation from 2014 to 2015. Additionally, there was a rapid decline in hepatitis B and C occurrences from 2015 to 2016. Moreover, there was almost constant prevalence from 2016 to 2019. Being male and age group 15–44 and age ≥ 65 were significantly associated with the chance of acquiring hepatitis B infection. Therefore, this can affect the more productive age at the household and zonal levels. Hence, our recommendation is to improve awareness about a method of transmission, increase resource mobilization such as immunization to that group, increase coverage of youth-friendly services in the health facilities and guidelines for screening and diagnosis should be implemented.

## Data Availability

All data analyzed during this study are included in this published article.

## Abbreviations

**HBsAg**: Hepatitis B surface antigen
**HBV**: Hepatitis B virus
**HCV**: Hepatitis C virus
**PCR**: Polymerase Chain Reaction
**RERC**: Research Ethics Review Committee
**TRHB**: Tigrai Regional Health Bureau
**WHO**: World health organization
